# Precision Medicine for More Oxygen (P4O2)—Study Design and First Results of the Long COVID-19 Extension

**DOI:** 10.3390/jpm13071060

**Published:** 2023-06-28

**Authors:** Nadia Baalbaki, Jelle M. Blankestijn, Mahmoud I. Abdel-Aziz, Jan de Backer, Somayeh Bazdar, Inés Beekers, Rosanne J. H. C. G. Beijers, Joop P. van den Bergh, Lizan D. Bloemsma, Harm Jan Bogaard, Job J. M. H. van Bragt, Vera van den Brink, Jean Paul Charbonnier, Merel E. B. Cornelissen, Yennece Dagelet, Elin Haf Davies, Anne M. van der Does, George S. Downward, Cornelis M. van Drunen, Debbie Gach, J. J. Miranda Geelhoed, Jorrit Glastra, Kornel Golebski, Irene H. Heijink, Judith C. S. Holtjer, Sebastiaan Holverda, Laura Houweling, John J. L. Jacobs, Renée Jonker, Renate Kos, Ramon C. J. Langen, Ivo van der Lee, Asabi Leliveld, Firdaus A. A. Mohamed Hoesein, Anne H. Neerincx, Lieke Noij, Johan Olsson, Marianne van de Pol, Simon D. Pouwels, Emiel Rolink, Michael Rutgers, Havva Șahin, Daphne Schaminee, Annemie M. W. J. Schols, Lisanne Schuurman, Gitte Slingers, Olie Smeenk, Brigitte Sondermeijer, Paul J. Skipp, Marisca Tamarit, Inge Verkouter, Roel Vermeulen, Rianne de Vries, Els J. M. Weersink, Marco van de Werken, Yolanda de Wit-van Wijck, Stewart Young, Esther J. Nossent, Anke H. Maitland-van der Zee

**Affiliations:** 1 Department of Pulmonary Medicine, Amsterdam UMC, 1105 AZ Amsterdam, The Netherlands; n.baalbaki@amsterdamumc.nl (N.B.);; 2 Amsterdam Institute for Infection and Immunity, 1105 AZ Amsterdam, The Netherlands; 3 Amsterdam Public Health, 1105 AZ Amsterdam, The Netherlands; 4 Department of Clinical Pharmacy, Faculty of Pharmacy, Assiut University, Assiut 71526, Egypt; 5 Fluidda, Groeningenlei 132, 2550 Kontich, Belgium; 6 ORTEC BV, Department of Health, Houtsingel 5, 2719 EA Zoetermeer, The Netherlands; 7 Department of Respiratory Medicine, Maastricht University Medical Centre, 6229 HX Maastricht, The Netherlands; 8 NUTRIM School of Nutrition and Translational Research in Metabolism, 6200 MD Maastricht, The Netherlands; 9 Department of Internal Medicine, Maastricht University Medical Centre, 6229 HX Maastricht, The Netherlands; 10 Department of Internal Medicine, VieCuri Medical Center, 5912 BL Venlo, The Netherlands; 11 Thirona, Toernooiveld 300, 6525 EC Nijmegen, The Netherlands; 12 Breathomix B.V., Bargelaan 200, 2333 CW Leiden, The Netherlands; 13 Aparito Netherlands B.V., Galileiweg 8, BioPartner 3 Building, 2333 BD Leiden, The Netherlands; 14 Department of Pulmonology, Leiden University Medical Center, 2333 ZA Leiden, The Netherlands; 15 Department of Environmental Epidemiology, Institute for Risk Assessment Sciences (IRAS), Utrecht University, 3584 CL Utrecht, The Netherlands; 16 Julius Center for Health Sciences and Primary Care, University Medical Center Utrecht, 3584 CG Utrecht, The Netherlands; 17 Department of Otorhinolaryngology, Amsterdam UMC, 1105 AZ Amsterdam, The Netherlands; 18 Quantib-U, Westblaak 106, 3012 KM Rotterdam, The Netherlands; 19 Department of Pulmonology, University Medical Center Groningen, 9700 RB Groningen, The Netherlands; 20 Department Pathology & Medical Biology, University Medical Center Groningen, 9700 RB Groningen, The Netherlands; 21 Longfonds, Stationsplein 125, 3818 LE Amersfoort, The Netherlands; 22 Department of Pulmonology, Spaarne Hospital, 2134 TM Hoofddorp, The Netherlands; 23 Department of Radiology, University Medical Center Utrecht and Utrecht University, 3508 GA Utrecht, The Netherlands; 24 Smartfish AS, Oslo Science Park, Gaustadalléen 21, 0349 Oslo, Norway; 25 Long Alliantie Nederland, Address Stationsplein 125, 3818 LE Amersfoort, The Netherlands; 26 Sodaq, Bussumerstraat 34, 1211 BL Hilversum, The Netherlands; 27 TopMD Precision Medicine Ltdincorporated, Southhampton SO45 3PN, UK; 28 Philips GmbH Innovative Technologies, 4646 AG Eindhoven, The Netherlands

**Keywords:** precision medicine, long COVID, infectious disease, prospective observational cohort study

## Abstract

**Introduction**: The coronavirus disease 2019 (COVID-19) pandemic has led to the death of almost 7 million people, however, with a cumulative incidence of 0.76 billion, most people survive COVID-19. Several studies indicate that the acute phase of COVID-19 may be followed by persistent symptoms including fatigue, dyspnea, headache, musculoskeletal symptoms, and pulmonary functional-and radiological abnormalities. However, the impact of COVID-19 on long-term health outcomes remains to be elucidated. **Aims**: The Precision Medicine for more Oxygen (P4O2) consortium COVID-19 extension aims to identify long COVID patients that are at risk for developing chronic lung disease and furthermore, to identify treatable traits and innovative personalized therapeutic strategies for prevention and treatment. This study aims to describe the study design and first results of the P4O2 COVID-19 cohort. **Methods**: The P4O2 COVID-19 study is a prospective multicenter cohort study that includes nested personalized counseling intervention trial. Patients, aged 40–65 years, were recruited from outpatient post-COVID clinics from five hospitals in The Netherlands. During study visits at 3–6 and 12–18 months post-COVID-19, data from medical records, pulmonary function tests, chest computed tomography scans and biological samples were collected and questionnaires were administered. Furthermore, exposome data was collected at the patient’s home and state-of-the-art imaging techniques as well as multi-omics analyses will be performed on collected data. **Results**: 95 long COVID patients were enrolled between May 2021 and September 2022. The current study showed persistence of clinical symptoms and signs of pulmonary function test/radiological abnormalities in post-COVID patients at 3–6 months post-COVID. The most commonly reported symptoms included respiratory symptoms (78.9%), neurological symptoms (68.4%) and fatigue (67.4%). Female sex and infection with the Delta, compared with the Beta, SARS-CoV-2 variant were significantly associated with more persisting symptom categories. **Conclusions**: The P4O2 COVID-19 study contributes to our understanding of the long-term health impacts of COVID-19. Furthermore, P4O2 COVID-19 can lead to the identification of different phenotypes of long COVID patients, for example those that are at risk for developing chronic lung disease. Understanding the mechanisms behind the different phenotypes and identifying these patients at an early stage can help to develop and optimize prevention and treatment strategies.

## 1. Introduction

The emergence of severe acute respiratory syndrome coronavirus 2 (SARS-CoV-2) in 2019, has led to a pandemic with major health and economic consequences for society. Human infection with SARS-CoV-2 can lead to coronavirus disease (COVID-19). According to the World Health Organization (WHO), on 4 April 2023, the global cumulative number of confirmed cases is over 0.7 billion and almost 7 million deaths. During the acute infection phase, SARS-CoV-2, causes symptoms including fever, chills, headache, muscle pain, cough and dyspnea that may be accompanied by hypoxemia, but also neurological or cardiovascular symptoms can occur [1,2]. This broad array of symptoms might be caused by the fact that SARS-CoV-2 can infect various cell types within the human body by the angiotensin-converting enzyme 2 (ACE2) receptor in presence of serine proteases including TMPRSS2 and TMPRSS4 [3]. ACE2 is expressed in many human cells including, but not limited to, respiratory, brain endothelial and vascular smooth muscle cells [4,5]. SARS-CoV-2 has challenged many existing social, economic, and scientific frameworks and processes including the speed of prevention and treatment of novel viral outbreaks. Today, innovative vaccine platforms help us in preventing a severe disease course of COVID-19. Nonetheless, a subset of people who have been infected with SARS-CoV-2 are struggling with persistent symptoms often referred to as long COVID [6,7].

Long COVID, post-COVID condition or post-acute sequelae of COVID-19 (PASC) is described as the presence of persistent symptoms following viral infection at 3 months after the onset of COVID-19 symptoms [8]. The reported incidence differs among performed studies, but is suspected to be at least 30% of all COVID-19 cases [9,10]. In the European region of the WHO, the prevalence of long COVID during the first two years of the pandemic is reported to be 17 million people at least [11]. Long COVID can occur after mild or severe acute COVID-19 [12]. Persistent symptoms for diagnosis can, among others, include fatigue, dyspnea, muscle weakness, insomnia, loss of taste and smell, headache and cognitive decline [13,14]. While other viral or bacterial infections are also known to sometimes cause long-term health symptoms, the mechanisms behind these long-term consequences of a SARS-CoV-2 infection are still not fully understood [15]. Hypothesized mechanisms include organ damage caused by excessive inflammation during acute COVID-19, autoimmunity due to molecular mimicry, the long-lasting presence of viral particles leading to ongoing inflammatory processes, clotting/coagulation issues, re-activation of neurotrophic pathogens, interactions with host microbiome/virome communities and dysfunctional brainstem/vagus nerve signaling [14,16,17]. Fortunately, the evidence supporting these and other mechanisms, driving long COVID, is rapidly increasing. For instance it was found that anticipating risk factors for long COVID can be type 2 diabetes, SARS-CoV-2 RNAemia, reactivation of latent Epstein-Barr virus and the presence of specific auto-antibodies [18]. Furthermore, it was demonstrated that the airways of post-COVID patients with radiological pulmonary abnormalities, showed distinct immune and proteomic profiles compared to healthy individuals [19]. Elevated protein concentrations were associated with epithelial tissue injury, apoptosis and tissue repair. Altered tryptophan absorption metabolism has also been proposed to be the main contributor to persisting symptoms in long COVID patients [20]. Tryptophan is an essential amino acid that can be degraded by the kynurenine pathway into kynurenine and was found to be upregulated during a SARS-CoV-2 infection. Metabolites of kynurenine may cause neurotoxicity which can lead to fatigue complaints in long COVID patients [21].

Long COVID is a complex condition, which is demonstrated by the current knowledge on this disease from a clinical perspective as well as the biological mechanisms driving it. Therefore, it emphasizes the need for a broader and more detailed understanding of the pathophysiological mechanisms of long COVID to reduce patient suffering and prevent new cases of long COVID. As mentioned, the reported symptoms that characterize long COVID patients are broad in spectrum and therefore require a research design that provides a broad overview of long COVID. We therefore set up the Precision Medicine for more Oxygen COVID-19 study (P4O2 COVID-19) in which we aim to assess if long COVID patients are at risk for developing chronic lung damage following a SARS-CoV-2 infection and to identify treatable traits and innovative personalized therapeutic strategies for prevention and treatment. In this paper, we present the design and first results of the P4O2 COVID-19 cohort. Innovative personalized therapeutic strategies can include drug repurposing of existing medications for specific long COVID patients, but can also be the discovery of a new target for drug discovery and that may ultimately serve as a personalized medicine strategy for long COVID patients with a similar phenotype.

## 2. Methods

### 2.1. Study Design and Participants

P4O2 COVID-19 is a multicenter prospective observational cohort study with a nested optional lifestyle intervention. This study was approved by the ethical board of the Amsterdam University Medical Center (UMC), reference number NL74701.018.20.

For the clinical P4O2 COVID-19 cohort, participants were recruited from post-COVID outpatient clinics from five different hospitals within The Netherlands. Participating hospitals reflected the academic and regional hospital populations across the country and included the both locations of the Amsterdam UMC, Leiden UMC, Spaarne Gasthuis and VieCuri Medisch Centrum. Inclusion criteria were a confirmed SARS-CoV-2 infection (quantitative polymerase chain reaction (PCR), serology tests or a COVID-19 Reporting and Data System (CO-RADS) score 4/5), a post-COVID outpatient clinic visit appointment, the ability to provide informed consent, aged 40–65 years, access to internet and understanding of the Dutch language. Exclusion criteria were the inability to provide informed consent, a terminal illness and participation in another study involving investigational or marketed products concomitantly or within four weeks prior to study entry or during the study.

**Figure 1:**
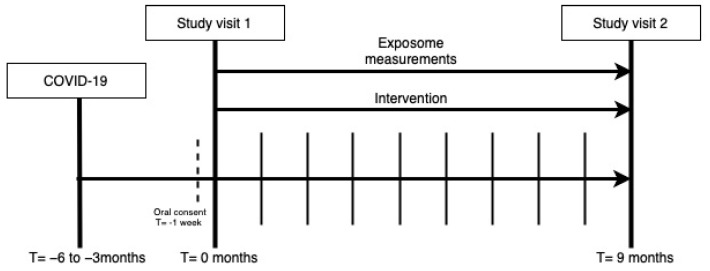
P4O2 COVID-19 participants were invited for a study visit at 3–6 and 12–15 months post-COVID-19. Between study visit 1 and 2, appointments have been made for exposome measurements and the optional intervention. Each bar between T = 0 and T = 9 months, represents 1 month during which the monthly questionnaire has been administered.

In The Netherlands, patients that suffer from persistent symptoms following mild or severe acute COVID-19 can be referred to an outpatient clinic where post-COVID care is provided by a multidisciplinary team of healthcare professionals. Patients that were hospitalized during acute COVID-19 are followed up 6 weeks after hospital discharge to decide if a further 3–6 month follow up appointment at an outpatient clinic is necessary. This decision is based on the persistence of symptoms that developed during the acute infection phase of COVID-19. Non-hospitalized patients that had an onset of acute COVID-19 and are still experiencing persistent symptoms, can be referred to the outpatient clinic by their general practitioner. In this cohort, we included patients from these outpatient clinics in participating hospitals. Included patients were screened from electronic hospital information system patient lists in the five participating centers that consisted of patients that were clinically followed up for COVID-19. Scheduled visits at the outpatient clinic included an appointment with a physician and physiotherapist as well as pulmonary function testing (spirometry, diffusing capacity of the lungs for carbon monoxide (DLCO)), non-contrast thorax computed tomography (CT) scan and blood withdrawal for laboratory measurements. Laboratory measurements included hemocytometry, blood differential test, renal function, electrolytes, glucose, proteins, liver function and other enzymes including N-terminal pro b-type natriuretic peptide (NT-PROBNP). CT scans will be analyzed with advanced automated quantitative imaging techniques. These will be performed on pulmonary tissue (including airway and lobar volumes, emphysema, airway wall thickness, air trapping), extra pulmonary tissue (including subcutaneous/visceral fat distribution, abdominal/psoas/spine muscles, bone mineral density) and vascular structures (including vascular volume, vascular fraction). P4O2 COVID-19 study visits were planned in parallel to the outpatient clinic visit at 3–6 months after infection and follow up study visits are planned 9 months later at 12–15 months after COVID-19 (Figure 1). In addition to these study visits, included patients were contacted to schedule home visits for exposome measurements. Finally, if the patient was willing to participate in the nested personalized lifestyle counseling intervention, the patient was also contacted for an intake appointment.

### 2.2. Study Visits

**Figure 2:**
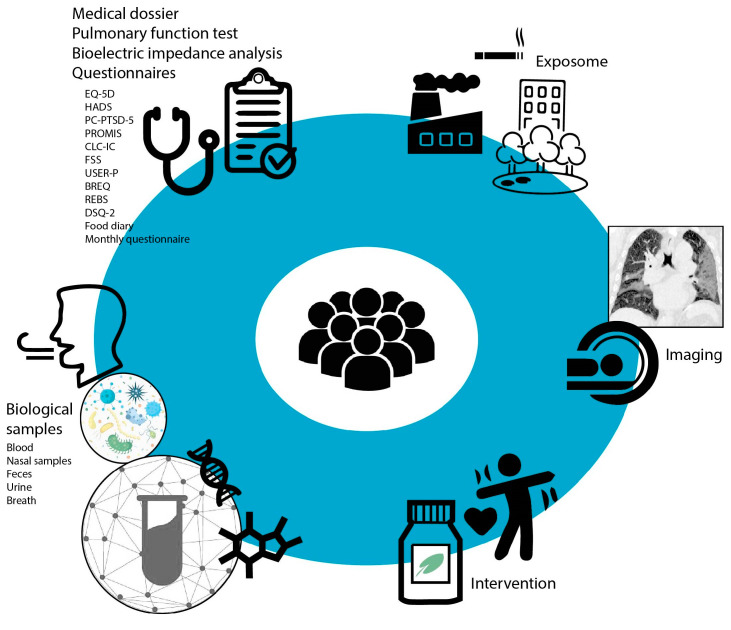
An overview of the data and sample collection during study visits and clinical follow-up of P4O2 COVID-19 patients. Parts of the figure were created by using pictures from The NOUN Project.

**Figure 3:**
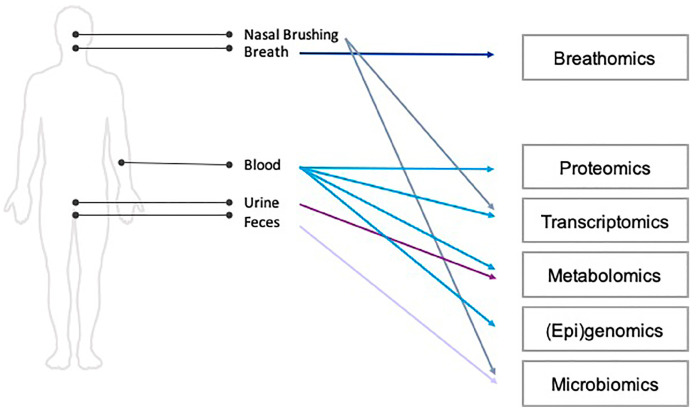
Omics analyses per type of collected biological sample from P4O2 COVID-19 study participants. Parts of the figure were created by using pictures from Servier Medical Art. Servier Medical Art by Servier is licensed under a Creative Commons Attribution 3.0 Unported License (https://creativecommons.org/licenses/by/3.0/, accessed on 3 July 2021).

**Figure A1:**
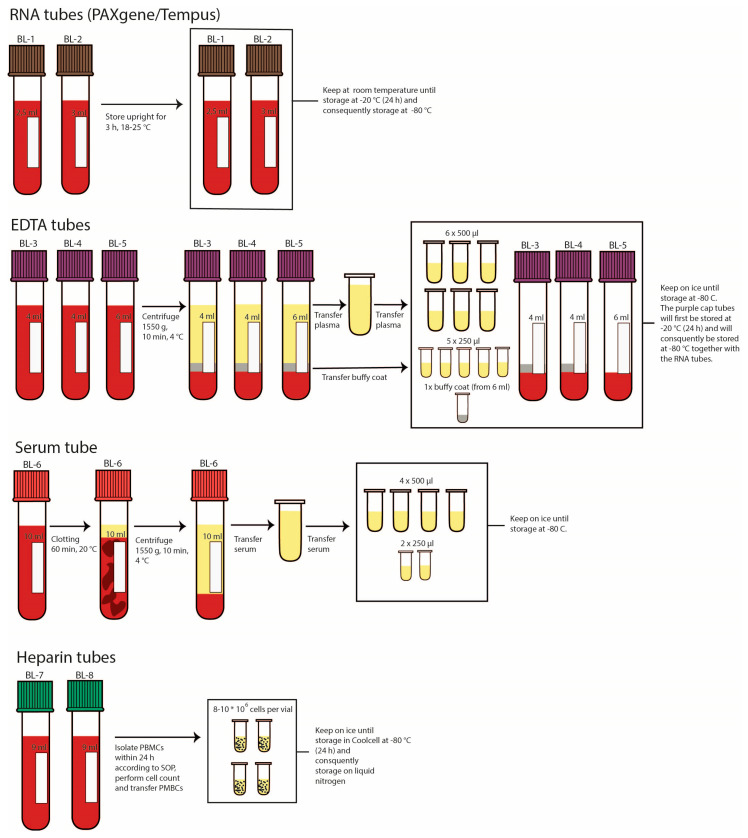
Processing and storage of collected blood samples.

**Figure A2:**
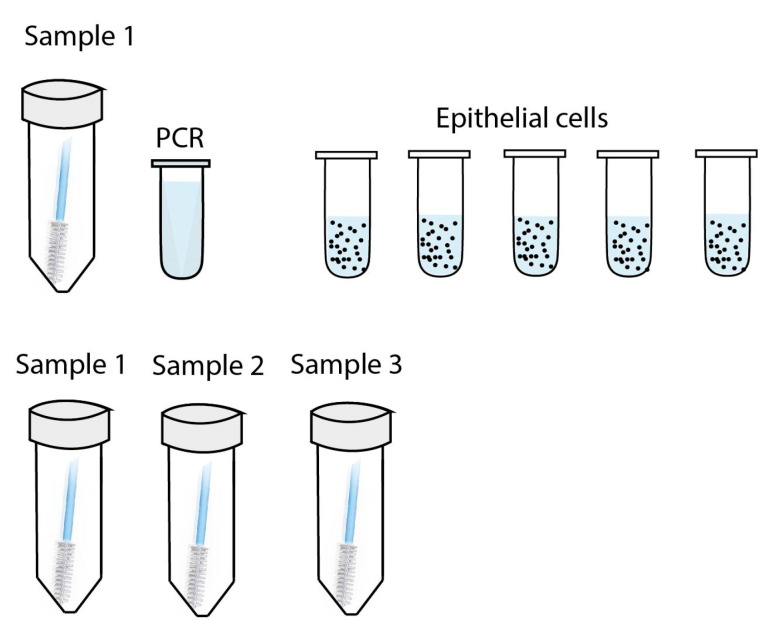
Processing and storage of collected nasal samples.

During the first study visit, written consent was obtained from the patient and baseline study characteristics concerning a participant’s health status prior to and during COVID-19 were assessed. During the first and second study visits details on general health and medication usage were collected from the electronic patient files, biological samples were collected, a bioelectric impedance analysis and real-time analysis of exhaled breath were performed and the patient was asked to fill in questionnaires (see Figure 2). Biological samples included exhaled breath, blood, nasal brushes, feces and urine. These samples will be used for immunological, nutrient and multi-omics analyses. See Figure 3 for an overview of the omics analyses that will be performed on the collected biological samples. Questionnaires were administered to describe health characteristics of patients from a physical, fatigue, cognitive, psychological, selfcare and participation perspective. A detailed description of the collected samples and administered questionnaires can be found in the Appendix A (Figure A1 and Figure A2).

### 2.3. Exposome

The exposome is defined as the sum of an individual’s lifetime exposures and how those exposures relate to health [22]. In the P4O2 COVID-19 cohort, personal exposome measurements were performed to investigate how environmental factors can contribute to the development of long-term symptoms in COVID-19 patients. Measurements were collected using four different devices: Sniffer Bike (Sodaq, Hilversum, Netherlands), Ultrasonic Personal Air Sampler (UPAS), silicone wristband, and the GARMIN activity tracker), each of which measures a different component of the exposome, see Appendix A for a detailed description of the used devices.

### 2.4. Intervention

All subjects were invited to participate in a lifestyle intervention, which consisted of personalized counselling on dietary quality and physical activity and/or nutritional support during the follow-up period of nine months. See Appendix A for a detailed description. In case subjects were not willing to participate, they continued the regular track of the study and were considered as control subjects for the intervention group.

### 2.5. Sample Size Determination

Prior to the start of this study, there was no published data available on long COVID related to pulmonary, extra-pulmonary manifestations and general health. Therefore, a power calculation was difficult based on known effect sizes. For some of the planned analyses, including microbiome and metabolomics, we could show that 100 participants would be sufficient. For the microbiome analyses, power calculations assuming a small effect size (ϕ > 0.05) to derive the parameters for the Dirichlet-multinomial distribution revealed an excellent power to detect an association (>95%) for a sample size of 100 participants [23]. For metabolomics, we have enough power to detect differences of 20% in targeted metabolites (approximately 630 metabolites using the BiocratesMxP^®^ Quant 500 kit) for 80% power at a false discovery rate < 0.05 for a sample size of 100 [24,25].

### 2.6. Analysis of Baseline and Long COVID Characteristics

Baseline characteristics were analyzed for the full study cohort. The total patient group was stratified into groups based on two long COVID outcome parameters. The first outcome parameter is based on the number of symptoms categories and the second parameter on pulmonary function test and radiological abnormalities (see detailed description below). Both the Chi Squared Test and Fisher’s Exact Test were used to identify statistically significant associations between one of these outcome parameters and the baseline characteristics. Associations were considered significant if *p* < 0.05.

#### 2.6.1. Classification of COVID-19 Severity and Variant

COVID-19 was classified as ambulatory, mild or severe based on the WHO ordinal scale for clinical improvement [26]. Criteria for mild disease included: hospitalized no oxygen therapy, oxygen by mask or nasal cannula. Severe disease included ventilated patients. The suspected COVID-19 variant was determined based on the date of infection. As no PCR test was performed to identify the virus variant, it was assumed that the patient was infected with the dominant variant at the time of infection [27].

#### 2.6.2. The Number and Classification of Persisting Symptoms

The number of persisting symptoms was primarily based on the baseline symptom questionnaire, which was taken between 0 and 1 months after the first study visit. This information was combined with the medical records in the hospital information system of the post-COVID outpatient clinic. Both the questionnaire and the appointment at the post-COVID outpatient clinic were between 3 and 6 months after a SARS-CoV-2 infection and therefore classified as ‘baseline’. The self-reported symptoms were checked for consistency with electronic patient files.

Persistent long COVID symptoms were then classified according to categories of symptoms including [28]:

- Fatigue
- Respiratory
- Neurological
- Cardiovascular
- Gastrointestinal
- Other

After classification of symptoms, subgroups were made based on the amount of present symptom categories, to obtain a better understanding of the involvement of multiple organ systems in relation to baseline characteristics of the study population.

#### 2.6.3. Pulmonary Function Test and Radiological Abnormalities

The presence of pulmonary function test and radiological abnormalities was based on CT scan and pulmonary function tests. The radiology reports of CT scans that were written by radiologists at the respective radiology departments of each participating center, were classified as abnormal if one or more of the following terms were included in the radiology report: consolidations/ground glass opacities, bronchiectasis, subpleural reticulation, honeycombing, lymphadenopathy, air trapping and dilated truncus pulmonalis. These long-term persisting radiographic abnormalities of COVID-19 were found on CT scans in other studies [6,29,30]. Pulmonary function testing was considered abnormal if forced vital capacity (FVC) and/or forced expiratory volume (FEV1) < 90% and/or DLCO < 70% and/or Tiffeneau-Pinelli index < 70%. The criteria for function test/radiological pulmonary abnormalities were consequently based on either or both abnormalities on the CT scan or in the pulmonary function test.

#### 2.6.4. Data Management and Statistical Analysis

All data is collected in compliance with the GDPR framework, with pseudonymized data and access limited on a need-to-know basis. Data from electronic case record files, the study visits exposomes and interventions are pseudonymously collected in LogiqScience and LogiqCare (ORTEC). R software (version 4.0.3; R Foundation for Statistical Computing, Vienna, Austria) was used to perform analyses and visualize data. Both the Chi Squared Test and Fisher’s Exact Test were used to identify statistically significant associations between two categorical variables and the odds ratio (OR). The choice of statistical test was dependent on the expected values. Student’s *t*-test or Mann-Whitney *U* was used to compare numerical variables, depending on the normality of the data. The significance threshold was set at 0.05 for all tests.

## 3. Results

**Figure 4:**
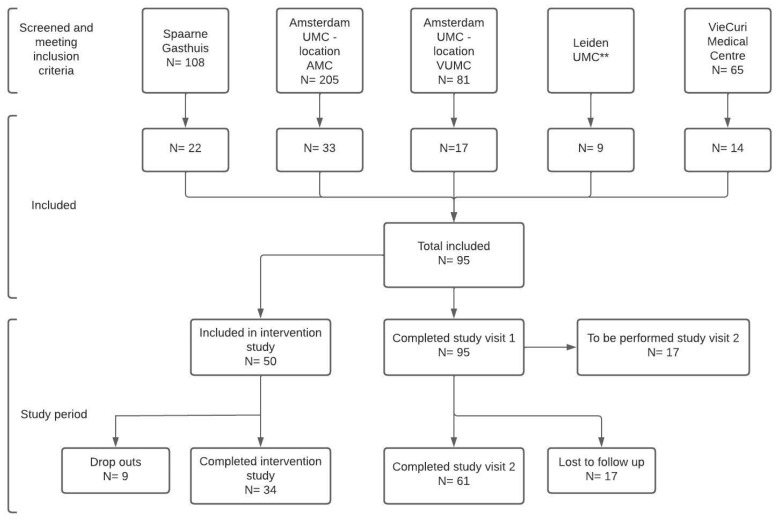
Screening and recruitment from five participating hospitals in P4O2 COVID-19. Numbers based on situation in April 2023. ** Leiden UMC medical doctors selected patients meeting the inclusion criteria and only forwarded patients to the research department when willing to participate in this study. Therefore, no list of the total eligible patients was obtained.

In total, 95 patients were enrolled between May 2021 and September 2022, of which 610 have already participated in the second study visit. Figure 4 provides an overview of the screening and recruitment process for P4O2 COVID-19 from the five participating hospitals within the Netherlands. Reasons for not including eligible patients differed between the first and second contact attempt. The first contact moment was to inform the patient about the study as well as to send the patient information folder, whereas the second contact was to obtain oral consent. For the first contact moment, most patients that did not give consent to receive the patient information folder could not be reached by telephone or email (22.6%) or did not provide a specific reason (19.2%). For the second contact, most frequent reasons for not including patients in the study included that they could not be reached by telephone or email (29.4%), did not provide a specific reason (17.6%) or experienced a lack of time (17.6%).

### 3.1. Baseline Characteristics and Long COVID Symptoms

The baseline characteristics of 95 participants are summarized in Table 1. The majority of the study participants were overweight (37.2%) or obese (52.1%). The most common comorbidities (prior to SARS-CoV-2 infection) are cardiovascular disease, diabetes and asthma. According to the WHO classification, most patients (64.8%) had mild COVID-19 infection. Most of the patients were hospitalized for COVID-19 (89.5%) of which about 30% were admitted to the ICU. The mean hospital duration was 8.5 days and the most common oxygen supplementation method was a nasal cannula. Nearly all patients received immunosuppressive medication, of which dexamethasone was most commonly prescribed. The most prevalent persisting symptoms were respiratory symptoms, which occurred in 78.9% of all patients, followed by persisting neurological (68.4%) and fatigue (67.4%) symptoms.

### 3.2. Long COVID Symptoms

**Figure 5:**
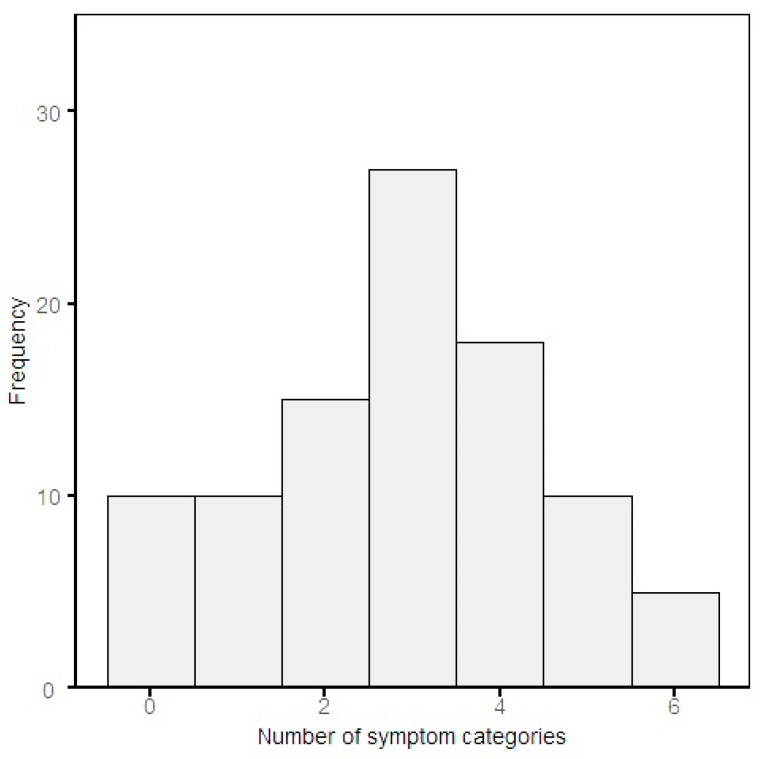
Number of symptom categories for each individual patient at baseline. Symptom categories includes fatigue, respiratory, neurological, cardiovascular, gastrointestinal and other.

The number of persisting symptoms was based on two sources: baseline symptom questionnaires and medical records. Consistency between the sources was checked for patients that had both sources available. For 68% of these patients, the symptoms that were mentioned in the baseline symptom questionnaire were also mentioned in the medical dossier. Most patients still experience symptoms from three symptom categories, of which respiratory, neurologic and fatigue symptoms were frequently reported (Figure 5 and Table 1).

The function test/radiological pulmonary abnormalities were derived from the radiological report on the performed CT scan and function test abnormalities were based on the performed pulmonary function testing. The most common radiological abnormalities were consolidations/ground glass opacities (N = 54, 56.8%). In Table 2, the CT scan abnormalities among study participants are shown. The most prevalent pulmonary function testing abnormality was FVC or FEV1 < 90% (N = 49, 54.4%). The number of patients that had at least one radiological or function test abnormality was 36 (46.2%). There were 42 patients (53.8%) who had abnormalities on both CT scan and pulmonary function testing.

Stratified baseline characteristics, for both long COVID outcome parameters, are shown in Table 3. For the number of symptoms according to the classification of symptoms into the described categories, the cut-off value for stratification was two symptom categories as this resulted in the most equal group sizes (see Figure 3). These two categories mostly included a combination of fatigue and respiratory symptom categories (Chi Squared Test: *OR =* 10.52, *p* < 0.001). The persistence of respiratory symptoms was not significantly associated with radiological or pulmonary function test abnormalities (Fisher’s Exact Test: *OR* = 1.70, *p* = 0.48). A significant number of female participants experienced more than two symptom categories (Chi Squared Test: *OR* = 0.25, *p* < 0.0019). The education levels, including secondary and vocational education, bachelor and master, differed significantly among participants with symptoms in two or fewer categories and more than two symptom categories *p =* 0.025. The comorbidities diabetes and cardiovascular disease were significantly more present amongst patients with fewer than two symptom categories (Chi Squared Test: *OR* = 0.24, *p* = 0.015 and 0.35, *p* = 0.027), respectively). Furthermore, compared to the Beta SARS-CoV-2 variant, the Delta variant is associated significantly with the presence of more than two symptom categories in this study (Chi Squared Test: *OR* = 3.54, *p* = 0.014). Additionally, a sensitivity analysis was performed to study the distribution of patient characteristics for patients without pre-existing lung disease (N = 74). All patients with pre-existing lung disease were excluded from the groups with and without presence of radiographic and pulmonary function test abnormalities, which did not result in relevant differences of baseline characteristics (Appendix A Table A1).

The questionnaire outcomes, see Table 4, showed that 75.9% of patients suffer from fatigue based on their FSS score, which is similar to the amount of people with reported fatigue symptoms. Furthermore, most respondents experienced problems in their daily activities and pain/discomfort sections. While 67.9% of the patients experienced problems in their daily activities based on the EQ-5D questionnaire, the USER-P showed that 26.5% of patients experienced restriction in their daily activities. Of all included patients that have been admitted to the hospital during acute COVID-19, 76.3% still experienced impairment in cognitive functioning.

## 4. Discussion

The aim of the Precision Medicine for more Oxygen COVID-19 study is to assess if long COVID patients are at risk for developing chronic lung damage following a SARS-CoV-2 infection and to identify treatable traits and innovative personalized therapeutic strategies for prevention and treatment of long COVID. Here, the baseline characteristics of the 95 study P4O2 COVID-19 participants were described and analyzed based on persisting symptom categories as well as pulmonary function test/radiological abnormalities. The main findings of this study are that most participants experience persisting symptoms at 3–6 months post-COVID of which respiratory, neurological fatigue symptoms are most common. Female sex and infection with the Delta, compared with the Beta, SARS-CoV-2 variant associated significantly with the presence of more than two symptom categories. Furthermore, the majority of subjects show signs of pulmonary function test/radiological damage. A major strength of this study is that a large amount of data was collected on each participating long COVID patient, ranging from a variety of biological samples to medical data and administered questionnaires that can contribute to a better understanding of the pathophysiology of this, currently, poorly understood heterogeneous condition.

According to this study, which was performed in post-COVID patients that were clinically followed-up for long COVID, the most prevalent symptoms at 3–6 months post-COVID-19 are respiratory and fatigue symptoms. This finding is in line with other studies performed internationally. In a German study that included patients after mild and moderate COVID-19, 61.9% of all patients reported persistent symptoms of which the most common were fatigue, sleep and respiratory problems [31]. The occurrence rate of these symptoms (N = 1027) was lower compared to the current study, in which 67.4% and 78.9% of all patients experienced fatigue or respiratory symptoms at baseline, respectively. An explanation for this difference could be that the participants of the P4O2 COVID-19 cohort were screened based on a confirmed SARS-CoV-2 infection rather than persisting symptoms or pulmonary function test/radiological abnormalities. A study performed in Italy found that 3 months after the onset of the first symptoms of COVID-19, 32% of all patients had one or two symptoms and 55% had three or more. The most common reported symptoms in this study included fatigue, dyspnea, joint pain and chest pain [7]. In the current study, 38% of all patients had two or fewer symptom categories and 62% of all patients experienced more than two symptom categories. In another study performed in the United States of America with hospitalized and non-hospitalized patients, the most prevalent symptoms at 3–4 months post-COVID in both patient groups were fatigue and dyspnea [32]. In a study where 6 month outcomes on long COVID symptoms were assessed in a group of patients that were discharged from the hospital for COVID-19, the most common symptoms were fatigue or muscle weakness (63%) [6]. According to Davis et al., the most frequently occurring symptom at 7 months post-infection is fatigue. Interestingly, persisting respiratory symptoms seem to decline over time [33], while during the acute phase of COVID-19 respiratory symptoms are reported more frequently than other symptoms including fatigue [34]. Neurological symptoms are also frequently reported among long COVID patients in this patient cohort, at 68.4%. Less is reported about the presence of neurological symptoms, compared to fatigue and respiratory symptoms. However, in a study of non-hospitalized COVID-19 survivors, that did not experience neurological complaints 6 months prior to this study (N = 52), neurological symptoms including tingling, cognitive dysfunction, headache, loss of taste and loss of smell were present at a comparable prevalence rate [35].

So, when comparing the persisting long COVID symptoms that were found in the current study to other studies performed in different countries around the world, the remaining symptoms seem to be in accordance with the ones found in our study based on Dutch patients suffering from long COVID.

Interestingly, female sex is associated with the presence of more symptom categories. While the number of symptom categories cannot be associated with increased severity of long COVID, in a number of studies female sex was found to be a risk factor for long COVID [36,37,38,39]. The comorbidities diabetes and cardiovascular disease associated with the presence of two or fewer symptom categories. Furthermore, the education levels differed significantly among participants with symptoms in two or fewer categories and more than two symptom categories. From the results, it can be concluded that these significant associations were not caused by the severity of COVID-19 and require further investigation.

The finding that fatigue is a frequently reported symptom was also confirmed by validated questionnaires. The mean FSS score exceeded the normal range in 75.9% of all patients that completed this questionnaire at baseline, which is comparable to the percentage of the self-reported/medical dossier-reported symptom fatigue. The DSQ-2 might provide us with additional insights into the fatigue that participants are experiencing. Furthermore, viral infections can lead to myalgic encephalomyelitis/chronic fatigue syndrome (ME/CFS), a neuroinflammation-linked condition that can be characterized by severe fatigue [40]. In a prospective observational study, post-COVID patients fulfilled the criteria for the diagnosis of ME/CFS [41]. The DSQ-2 may also demonstrate whether patients suffering from persistent fatigue symptoms, meet the diagnostic criteria for ME/CFS.

Other questionnaires pointed out that long COVID patients suffer from psychological problems including anxiety (39.3%), depression (29.6%) and posttraumatic stress symptoms (PTSD) (27.7%). In a prospective cohort study that was conducted in Italy, among 767 patients that were hospitalized for COVID-19, it was found that at 4 months after hospital discharge, 17.2% of all patients had PTSD symptoms [42]. The same study also reported a limited mobility score as was similarly found according to the EQ-5D mobility domain in the current study [42]. A Northern European observational study on 247,249 individuals who were diagnosed with COVID-19, found that patients that were admitted to the hospital for more than 7 days were persistently at higher risk for symptoms of depression and anxiety [43]. However, the Trimbos Institute found an increased number of people that reported anxiety and depression symptoms during the COVID-19 pandemic among the Dutch population in general [44]. Interestingly, according to the EQ-5D, people experience problems with daily activities including work, social, housekeeping or leisure activities (67.9%) whereas according to the USER-P questionnaire only 26.5% patients experience restrictions in their daily activities. A reason for this observation could be that for the EQ-5D any degree of experienced problems in daily activities is regarded as a problem, whereas in the USER-P restriction in daily activities is defined as 60% restriction in daily activities based on multiple questions. Therefore, in the USER-P a patient is less likely to report restrictions in overall daily activities compared to the EQ-5D question on daily activities. Further research into the impact of long COVID on restrictions/problems in the ability to perform daily activities is needed to clarify these findings.

The most frequently found comorbidities were cardiovascular disease, asthma and diabetes. Additionally, 52.1% of all subjects were obese, which is higher compared to the percentage of obesity within the Dutch population [45]. It is already known that chronic conditions including hypertension are associated with increased severity of acute COVID-19 [46]. In a longitudinal study on 309 patients that were followed from their initial diagnosis to 2–3 months later, type 2 diabetes was found to be an anticipating risk factor for the development of long COVID [18]. In another longitudinal prospective cohort study in adults with a confirmed SARS-CoV-2 infection, 35.5% of patients with hypertension or diabetes experienced ongoing symptoms [12].

During the patient inclusion period of P4O2 COVID-19, several SARS-CoV-2 strains were circulating. A study limitation is that there is no available data on the SARS-CoV-2 variant that study participants were infected with. Based on pathogen surveillance data of the Dutch National Institute for Health and Environment, the dominating strain during the inclusion period was Delta SARS-CoV-2. At the start of patient inclusion, Beta/Gamma variants were most frequently observed, while currently, Omicron SARS-CoV-2 variants are most frequently observed [27]. In the current study, the Delta SARS-CoV-2 variant associated significantly with the presence of more than two symptom categories compared to the Beta SARS-CoV-2 variant. The other SARS-CoV-2 variants, Gamma and Omicron, were less common in this patient population to provide sufficient power to detect significant differences. This finding, however, cannot be used for conclusions regarding possible associations between coronavirus variants and long COVID incidence. In a large case-control observational study performed in the UK during the Omicron period compared with the Delta period, it was shown that Delta SARS-CoV-2 is associated with increased risk of long COVID [47]. Although it is suggested that Omicron may lead to larger absolute numbers of long COVID patients, since its transmission rate is higher [47].

For this study, symptom categories were based on monthly questionnaires and medical dossiers. For some patients, additional symptoms were mentioned in either the baseline symptom questionnaire or the medical dossier. Inconsistencies are to be expected with the fluctuating nature of long COVID symptoms as well as the physician’s different interview techniques and reporting styles in medical records. Furthermore, this implies that for patients from whom no baseline symptom questionnaire data was available, symptoms were solely based on the medical dossier and therefore a small percentage of symptoms may be missing which can be regarded as a study limitation. However, for patients that have both sources available, the medical dossier overall strengthens the self-reported symptoms in the baseline symptom questionnaire, because of the high resemblance.

The most commonly found radiological abnormalities were consolidations/ground glass opacities, which is in accordance with other studies [6,29,48]. Interestingly, in the current study, no statistically significant association was found between patients that had persisting respiratory symptoms and radiological/pulmonary abnormalities, which may be related to the limited number of patients. Whereas, in a study by Lehmann et al., function test and radiological pulmonary abnormalities were seen more frequently in patients with long-lasting respiratory symptoms [49]. Baseline characteristics were not significantly different amongst participants with or without presence of radiological/pulmonary function test abnormalities. This outcome may be affected by the relatively small group size of study participants without these abnormalities. Furthermore, the exclusion of patients with pre-existing lung disease from these analyses did not result in other relevant findings. A study limitation, however, is that it is unknown if patients without pre-existing pulmonary disease showed signs of radiological/pulmonary function test prior to COVID-19. The definition of radiological abnormalities was broad in this study. Therefore, future study aims are to elaborate on the clinical significance of specific radiological abnormalities. Pulmonary function test abnormalities were based on arbitrary cut-off values to pre-select patients with possible pulmonary function impairment. The clinical relevance of these findings remains as a future study aim.

## 5. Conclusions

The current study shows that P4O2 COVID-19 patients have pulmonary function test/radiological abnormalities and persisting symptoms, of which respiratory and fatigue are most prominently reported at 3–6 months post-COVID-19. Baseline characteristics including female sex and infection with the Delta, compared with the Beta, SARS-CoV-2 variant associated significantly with a higher number of persisting symptom categories. In contrast to most other studies on long COVID, the current study focuses on long COVID patients that experience persisting symptoms and are therefore clinically followed up which can lead to valuable new insights on long COVID patients specifically. This difference in patient selection compared to other studies could be an explanation for the higher observed number of persisting symptoms. Compared to studies that included post-COVID patients with a confirmed SARS-CoV-2 infection, but not necessarily with long COVID symptoms, the type of persisting symptoms and pre-existing conditions generally seem to be in accordance with each other. The implications of these findings on persisting symptoms and pulmonary function test/radiological abnormalities for treatment strategies, remain to be elucidated. The large number of data that is currently being collected longitudinally, during 9 months of follow-up, will provide a broad and detailed context of patients suffering from long COVID. As described, the nested personalized counseling intervention within this study may demonstrate its modifiable potential on remaining damage and progression to chronic lung damage. Mapping of the exposome may provide answers to which extent social and environmental factors impact long COVID symptoms. Additionally, the state-of-the-art advanced automated quantitative imaging techniques that will be applied to CT scans of P4O2 COVID-19 will provide more details on observed and/or novel pulmonary function test/radiological abnormalities and their relation to long COVID symptoms. The analyzed parameters will therefore lead to a detailed insight of how COVID-19 has affected the various types of tissues in and around the lungs, on both an anatomical and functional level. Immunological analyses and multi-omics analyses ((epi)genome, transcriptome, proteome, metabolome, microbiome as well as breathomics) could reveal novel mechanisms driving these long COVID symptoms and pulmonary function test/radiological abnormalities that can be used to find new targets for drug development or repurposing. In parallel to the P4O2 COVID19 project, in vitro studies with novel ‘on-chip’ models will be performed that will examine if and how external-and internal factors can modify the risk of long COVID-19. Following these first baseline study results, the combined efforts of the P4O2 consortium in a unique study population of long COVID patients may contribute to the identification of treatable traits and innovative personalized therapeutic strategies for the prevention and treatment of long COVID.

## Figures and Tables

**Table 1:** Baseline characteristics of P4O2 COVID-19 study participants, N = 95. In case of missing data, it will be indicated of how many patients the data is available. Categorical variables are described as n (% of n), and continuous variables as median (interquartile range, (IQR)) or mean ± standard deviation depending on normality. COPD: chronic obstructive pulmonary disease.

	Data (N = 95)
General	
Age	54.15 ± 6.18
Female	47 (49.5%)
Smoking status	
Current smoker	4 (4.2%)
Ex-smoker	51 (53.7%)
Never smoker	40 (42.1%)
BMI	
Normal	10/94 (10.6%)
Overweight	35/94 (37.2%)
Obese	49/94 (52.1%)
Ethnicity	
Caucasian	66/86 (76.7%)
African	8/86 (9.3%)
Asian	3/86 (3.5%)
Latin-American	3/86 (3.5%)
Other	6/86 (7.0%)
Level of education	
Secondary education	19/79 (24.1%)
Vocational education	33/79 (41.8%)
Bachelor	19/79 (24.1%)
Master	8/79 (10.1%)
Current comorbidities	
Asthma	16/94 (17.0%)
COPD	6/94 (6.4%)
Cardiovascular disease	27/93 (29.0%)
Heart failure	6/94 (6.4%)
Deep vein thrombosis and pulmonary embolism	10/94 (10.6%)
Diabetes	15/94 (16.0%)
Hepatic disease	6/93 (6.5%)
Renal Failure	7/92 (7.6%)
Interstitial lung disease	2/94 (2.1%)
Neurological disorders	3/94 (3.2%)
Rheumatic diseases	9/94 (9.6%)
Acute COVID-19 characteristics	
No hospitalization	10 (10.5%)
Hospitalization	85 (89.5%)
Intensive care admission	27 (28.4%)
Duration of hospitalization	84; 8.50 (6.00, 16.00)
Thrombosis	14/91 (15.4%)
Pulmonary embolism	15/92 (16.3%)
WHO disease Severity (mild)	10 (10.5%)
WHO disease Severity (moderate)	61 (64.2%)
WHO disease Severity (severe)	24 (25.3%)
Oxygen supplementation and ventilation	
Nasal cannula	40 (42.1%)
Non-rebreathing mask	16 (16.8%)
Non-invasive ventilation	5 (5.3%)
Invasive ventilation	19 (20.0%)
Vaccination status	
No	28 (29.5%)
Yes, 1 dose	24 (25.3%)
Yes, 2 or more doses	43 (45.3%)
Pharmacological treatment during acute COVID-19	
Antivirals	1/75 (1.3%)
Convalescent plasma	0/45 (0.0%)
Antibiotics	0/77 (0.0%)
Immunosuppressives	78/83 (94.0%)
Other	74/81 (91.4%)
Rest complaints	
Fatigue	64 (67.4%)
Respiratory	75 (78.9%)
Neurological	65 (68.4%)
Cardiovascular	25 (26.3%)
Gastrointestinal	26 (27.4%)
Other	18 (18.9%)
Dominant virus strains	
Beta	27 (28.4%)
Gamma	14 (14.7%)
Delta	43 (45.3%)
Omicron	11 (11.6%)
Blood tests	
Hemoglobin (mmol/L)	68; 8.70 (8.30, 9.50)
Hematocrit (L/L)	58; 0.42 (0.40, 0.46)
Thrombocytes (10^9^/L)	62; 259.00 (206.50, 295.25)
Leukocytes (10^9^/L)	62; 6.70 (5.62, 8.38)
Eosinophiles (10^9^/L)	55; 0.12 (0.08, 0.20)
Basophiles (10^9^/L)	54; 0.04 (0.03, 0.06)
Neutrophiles (10^9^/L)	49; 3.65 (2.98, 4.62)
Lymphocytes (10^9^/L)	54; 2.04 (1.70, 2.54)
Monocytes (10^9^/L)	54; 0.55 (0.45, 0.70)
Creatinine (μmol/L)	63; 79.00 (66.00, 88.00)
EGFR (mL/min/1.73 m^2^)	63; 89.00 (72.50, 90.00)
ALAT (U/L)	56; 25.50 (18.75, 35.25)
ASAT (U/L)	53; 22.00 (19.00, 27.00)
LDH (U/L)	55; 198.00 (168.00, 234.50)
CRP (mg/L)	55; 2.20 (1.30, 6.00)
Glucose (mmol/L)	39; 5.70 (5.25, 6.45)
Creatine Phosphokinase (U/L)	34; 89.50 (66.25, 179.00)

**Table 2:** Presence abnormalities on CT scans of study participants, N = 95.

	Data (N = 95)
Abnormalities	
Ground glass opacities/consolidations	54 (56.8%)
Bronchiectasis	19 (20.0%)
Subpleural reticulation	23 (24.2%)
Honeycombing	2 (2.1%)
Lymphadenopathy	9 (9.5%)
Air trapping	10 (10.5%)

**Table 3:** Baseline characteristics of P4O2 COVID-19 study participants, N = 95. Patients were classified according to the number of persistent symptom categories (left) or the presence of radiological/radiological abnormalities. In case of missing data, it will be indicated of how many patients the data is available. Categorical variables are described as n (% of n), and continuous variables as median (interquartile range, (IQR)) or mean ± standard deviation depending on normality. BMI: Body Mass Index. COPD: Chronic obstructive pulmonary disease.

	Symptoms in 2 or Fewer Categories (N = 35)	Symptoms in More than 2 Categories (N = 60)	No Pulmonary Function Test/Radiological Abnormalities (N = 14)	Pulmonary Function Test/ Radiological Abnormalities (N = 78)
General				
Age (Years)	53.66 ± 6.24	54.43 ± 6.18	51.64 ± 5.62	54.56 ± 6.13
Female	10 (28.6%)	37 (61.7%)	9 (64.3%)	36 (46.2%)
Smoking status				
Current smoker	1 (2.9%)	3 (5.0%)	0 (0.0%)	4 (5.1%)
Ex-smoker	17 (48.6%)	34 (56.7%)	9 (64.3%)	40 (51.3%)
Never smoker	17 (48.6%)	23 (38.3%)	5 (35.7%)	34 (43.6%)
BMI				
Normal	3/34 (8.8%)	7 (11.7%)	2 (14.3%)	8/77 (10.4%)
Overweight	14/34 (41.2%)	21 (35.0%)	2 (14.3%)	32/77 (41.6%)
Obese	17/34 (50.0%)	32 (53.3%)	10 (71.4%)	37/77 (48.1%)
Ethnicity				
Caucasian	19/28 (67.9%)	47/58 (81.0%)	13 (92.9%)	52/69 (75.4%)
African	3/28 (10.7%)	5/58 (8.6%)	0 (0.0%)	6/69 (8.7%)
Asian	1/28 (3.6%)	2/58 (3.4%)	0 (0.0%)	3/69 (4.3%)
Latin-American	1/28 (3.6%)	2/58 (3.4%)	0 (0.0%)	3/69 (4.3%)
Other	4/28 (14.3%)	2/58 (3.4%)	1 (7.1%)	5/69 (7.2%)
Level of education				
Secondary education	7/26 (26.9%)	12/53 (22.6%)	0/13 (0.0%)	18/63 (28.6%)
Vocational education	7/26 (26.9%)	26/53 (49.1%)	7/13 (53.8%)	24/63 (38.1%)
Bachelor	6/26 (23.1%)	13/53 (24.5%)	5/13 (38.5%)	14/63 (22.2%)
Master	6/26 (23.1%)	2/53 (3.8%)	1/13 (7.7%)	7/63 (11.1%)
Current comorbidities				
Asthma	3 (8.6%)	13/59 (22.0%)	2/13 (15.4%)	13 (16.7%)
COPD	0 (0.0%)	6/59 (10.2%)	0/13 (0.0%)	5 (6.4%)
Cardiovascular disease	15 (42.9%)	12/58 (20.7%)	2/13 (15.4%)	25/77 (32.5%)
Heart failure	3 (8.6%)	3/59 (5.1%)	0/13 (0.0%)	6 (7.7%)
Deep vein thrombosis and pulmonary embolism	3 (8.6%)	7/59 (11.9%)	1/13 (7.7%)	8 (10.3%)
Diabetes	10 (28.6%)	5/59 (8.5%)	0/13 (0.0%)	14 (17.9%)
Hepatic disease	4/34 (11.8%)	2/59 (3.4%)	0/13 (0.0%)	6/77 (7.8%)
Renal Failure	4/33 (12.1%)	3/59 (5.1%)	0/13 (0.0%)	6/76 (7.9%)
Interstitial lung disease	0 (0.0%)	2/59 (3.4%)	0/13 (0.0%)	2 (2.6%)
Neurological disorders	2 (5.7%)	1/59 (1.7%)	0/13 (0.0%)	3 (3.8%)
Rheumatic diseases	1 (2.9%)	8/59 (13.6%)	2/13 (15.4%)	7 (9.0%)
Acute COVID-19 characteristics				
No hospitalization	2 (5.7%)	8 (13.3%)	5 (35.7%)	4 (5.1%)
Hospitalization	33 (94.3%)	52 (86.7%)	9 (64.3%)	74 (94.9%)
Intensive care admission	13 (37.1%)	14 (23.3%)	2 (14.3%)	24 (30.8%)
Duration of hospitalization	33; 12.00 (7.00, 21.00)	51; 8.00 (4.50, 16.00)	9; 7.00 (3.00, 8.00)	73; 10.00 (6.00, 16.00)
Thrombosis	8/34 (23.5%)	6/57 (10.5%)	2/13 (15.4%)	12/75 (16.0%)
Pulmonary embolism	5/34 (14.7%)	10/58 (17.2%)	1/13 (7.7%)	14/76 (18.4%)
WHO disease Severity (mild)	2 (5.7%)	8 (13.3%)	5 (35.7%)	4 (5.1%)
WHO disease Severity (moderate)	23 (65.7%)	38 (63.3%)	8 (57.1%)	52 (66.7%)
WHO disease Severity (severe)	10 (28.6%)	14 (23.3%)	1 (7.1%)	22 (28.2%)
Oxygen supplementation and ventilation				
Nasal cannula	14 (40.0%)	26 (43.3%)	4 (28.6%)	36 (46.2%)
Non-rebreathing mask	7 (20.0%)	9 (15.0%)	3 (21.4%)	12 (15.4%)
Non-invasive ventilation	2 (5.7%)	3 (5.0%)	0 (0.0%)	5 (6.4%)
Invasive ventilation	8 (22.9%)	11 (18.3%)	1 (7.1%)	17 (21.8%)
Vaccination status				
No	11 (31.4%)	17 (28.3%)	4 (28.6%)	23 (29.5%)
Yes, 1 dose	11 (31.4%)	13 (21.7%)	4 (28.6%)	20 (25.6%)
Yes, 2 or more doses	13 (37.1%)	30 (50.0%)	6 (42.9%)	35 (44.9%)
Pharmacological treatment during acute COVID-19				
Antivirals	0/29 (0.0%)	1/46 (2.2%)	0/7 (0.0%)	1/66 (1.5%)
Convalescent plasma	0/17 (0.0%)	0/28 (0.0%)	0/5 (0.0%)	0/39 (0.0%)
Antibiotics	0/31 (0.0%)	0/46 (0.0%)	0/8 (0.0%)	0/67 (0.0%)
Immunosuppressives	32/33 (97.0%)	46/50 (92.0%)	8/9 (88.9%)	68/72 (94.4%)
Other	30/32 (93.8%)	44/49 (89.8%)	9/9 (100.0%)	63/70 (90.0%)
Rest complaints				
Fatigue	7 (20.0%)	57 (95.0%)	10 (71.4%)	52 (66.7%)
Respiratory	18 (51.4%)	57 (95.0%)	10 (71.4%)	63 (80.8%)
Neurological	11 (31.4%)	54 (90.0%)	10 (71.4%)	53 (67.9%)
Cardiovascular	1 (2.9%)	24 (40.0%)	3 (21.4%)	21 (26.9%)
Gastrointestinal	1 (2.9%)	25 (41.7%)	5 (35.7%)	20 (25.6%)
Other	2 (5.7%)	16 (26.7%)	4 (28.6%)	13 (16.7%)
Dominant virus strains				
Beta	15 (42.9%)	12 (20.0%)	1 (7.1%)	25 (32.1%)
Gamma	5 (14.3%)	9 (15.0%)	3 (21.4%)	11 (14.1%)
Delta	11 (31.4%)	32 (53.3%)	7 (50.0%)	34 (43.6%)
Omicron	4 (11.4%)	7 (11.7%)	3 (21.4%)	8 (10.3%)
Blood tests				
Hemoglobin (mmol/L)	24; 9.05 (8.38, 9.60)	44; 8.55 (8.28, 8.93)	9; 8.60 (8.30, 9.50)	57; 8.70 (8.30, 9.50)
Hematocrit (L/L)	19; 0.46 (0.43, 0.47)	39; 0.42 (0.40, 0.44)	7; 0.42 (0.40, 0.44)	49; 0.42 (0.41, 0.46)
Thrombocytes (10^9^/L)	22; 246.50 (178.00, 273.75)	40; 265.00 (236.75, 296.00)	7; 265.00 (235.00, 302.00)	53; 258.00 (206.00, 293.00)
Leukocytes (10^9^/L)	22; 7.15 (6.12, 8.88)	40; 6.10 (5.50, 7.90)	7; 7.00 (5.85, 7.80)	53; 6.80 (5.60, 8.50)
Eosinophiles (10^9^/L)	19; 0.15 (0.10, 0.18)	36; 0.11 (0.08, 0.19)	7; 0.10 (0.08, 0.20)	46; 0.13 (0.08, 0.20)
Basophiles (10^9^/L)	18; 0.04 (0.03, 0.07)	36; 0.04 (0.02, 0.06)	7; 0.05 (0.02, 0.08)	45; 0.04 (0.03, 0.06)
Neutrophiles (10^9^/L)	15; 3.89 (3.44, 4.50)	34; 3.57 (2.69, 4.59)	6; 3.70 (3.45, 4.32)	41; 3.85 (2.98, 4.83)
Lymphocytes (10^9^/L)	18; 2.39 (1.96, 3.14)	36; 1.94 (1.55, 2.39)	7; 1.90 (1.66, 2.50)	45; 2.07 (1.70, 2.56)
Monocytes (10^9^/L)	18; 0.69 (0.52, 0.85)	36; 0.50 (0.44, 0.60)	7; 0.59 (0.53, 0.60)	45; 0.54 (0.46, 0.71)
Creatinine (μmol/L)	23; 84.00 (74.50, 101.50)	40; 73.00 (65.75, 83.25)	8; 70.50 (64.75, 76.50)	53; 81.00 (68.00, 89.00)
EGFR (mL/min/1.73 m^2^)	23; 87.00 (69.00, 90.00)	40; 90.00 (75.25, 90.00)	8; 88.50 (83.50, 90.00)	53; 89.00 (72.00, 90.00)
ALAT (U/L)	19; 29.00 (20.50, 35.50)	37; 23.00 (18.00, 35.00)	6; 21.50 (17.25, 28.00)	48; 24.50 (19.75, 36.25)
ASAT (U/L)	18; 23.00 (18.75, 25.50)	35; 22.00 (19.50, 27.00)	6; 21.00 (20.00, 23.50)	45; 22.00 (18.00, 27.00)
LDH (U/L)	18; 200.50 (182.00, 218.75)	37; 194.00 (161.00, 242.00)	6; 173.00 (170.00, 197.75)	47; 202.00 (168.00, 237.00)
CRP (mg/L)	17; 3.20 (2.00, 6.00)	38; 2.00 (1.30, 6.00)	7; 2.40 (1.50, 7.00)	46; 2.10 (1.33, 6.00)
Glucose (mmol/L)	14; 5.90 (5.32, 6.80)	25; 5.50 (5.20, 6.30)	2; 5.00 (4.75, 5.25)	35; 5.70 (5.35, 6.45)
Creatine Phosphokinase (U/L)	11; 115.00 (69.50, 221.00)	23; 86.00 (66.50, 123.50)	2; 91.00 (88.50, 93.50)	30; 84.00 (66.00, 179.00)
NTProBNP (ng/L)	17; 50.70 (50.00, 82.00)	33; 50.70 (50.00, 99.90)	7; 75.00 (50.70, 109.20)	41; 50.70 (50.00, 87.00)

**Table 4:** Results from each individual questionnaire from the baseline visit. Scores are shown as mean ± standard deviation for the PROMIS questionnaire and median (IQR) for other questionnaires.

	Score	# Abnormal (%)
Physical function		
PROMIS	82; 28.72 ± 8.03	14/82 (17.1%)
Fatigue		
FSS	87; 5.56 (4.17, 6.28)	66/87 (75.9%)
Cognitive functioning		
CLC-IC	80; 5.00 (2.00, 8.00)	61/80 (76.3%)
Psychological functioning		
HADS anxiety	28; 4.00 (3.00, 10.25)	11/28 (39.3%)
HADS depression	27; 5.00 (1.50, 8.00)	8/27 (29.6%)
PC-PTSD-5	83; 1.00 (0.00, 2.00)	23/83 (27.7%)
Self care		
EQ-5D, mobility	84; 1.00 (1.00, 3.00)	36/84 (42.7%)
EQ-5D, self-care	84; 1.00 (1.00, 1.00)	13/84 (15.5%)
EQ-5D, daily activities	84; 2.00 (1.00, 3.00)	57/84 (67.9%)
EQ-5D, pain/discomfort	84; 2.00 (1.00, 3.00)	55/84 (65.5%)
EQ-5D, anxiety/depression	83; 1.00 (1.00, 2.00)	30/83 (36.1%)
**Participation**		
USER-P	83; 80.00 (60.00, 96.82)	22/83 (26.5%)

## Data Availability

The data presented in this study are available on request to the corresponding author. The data are not publicly available due to agreements made by the consortium, that only allow access by each consortium partner to specific data that answers their pre-specified research questions. A request for access to data by organizations outside of the consortium can be submitted to the P4O2 Data Committee (via p4o2@amsterdamumc.nl) and the research will need to be performed in collaboration with one of the P4O2 consortium partners.

## Abbreviations

SARS-CoV-2	severe acute respiratory syndrome coronavirus 2
COVID-19	coronavirus disease
WHO	World Health Organization
ACE2	angiotensin-converting enzyme 2
PASC	post-acute sequelae of COVID-19
P4O2 COVID-19	Precision Medicine for more Oxygen– COVID-19 study
UMC	University Medical Center
CO-RADS	COVID-19 Reporting and Data System
(q)PCR	(quantitative) polymerase chain reaction
DLCO	diffusing capacity of the lungs for carbon monoxide
CT	computed tomography
NT-PROBNP	N-terminal pro b-type natriuretic peptide
eNose	electronic nose
VOCs	volatile organic compounds
FSS	Fatigue Severity Scale
DSQ-2	DePaul Symptom Questionnaire 2
ME	myalgic encephalomyelitis
CFS	chronic fatigue syndrome
HADS	Hospital Anxiety and Depression Scale
USER-P	Utrecht Scale for Evaluation of Rehabilitation Participations
CLC-IC	Checklist for cognitive consequences after an ICU
ICU	intensive care unit
EQ5D	EuroQol 5D-5L
PROMIS	Patient-Reported outcome measurements information system
PC-PTSD-5	Primary Care PTSD Screen for DSM-5
BREQ-2	Behavioral Regulation in Exercise Questionnaire-2
REBS	Regulation of Eating Behaviors Scale
UPAS	Ultrasonic Personal Air Sampler
PM	particulate matter
EPA	eicosapentaenoic acid
DHA	docosahexaenoic acid
FVC	forced vital capacity
FEV1	forced expiratory volume
OR	odds ratio
IQR	interquartile range

## Appendix A

Study visits
Biological sample collection and measurements

Biological samples included exhaled breath, blood, nasal brushes, feces and urine. These samples will be used for immunological, nutrient and multi-omics analyses. See Figure 3 for an overview of the omics analyses that will be performed on the collected biological samples. Prior to these study visits, patients were asked to fill in a food diary and collect two feces samples that were collected at the beginning of each study visit. Two feces samples were stored in 15 mL feces collection tubes at −20 °Celsius (C) until further microbiome analysis. Blood was withdrawn by venipuncture in serum, EDTA, heparine (BD Vacutainer^®^) and RNA tubes (Tempus and PAXgene). An overview of the processing steps until storage of serum, EDTA plasma, blood peripheral mononuclear cells, buffy coat and whole blood, are shown in Appendix A Figure A1. Stored blood samples will be used for immunological, nutrient as well as transcriptome, metabolome, (epi)genome and proteome analyses. Nasal samples were taken with a Cytobrush Plus (Cooper Surgical) and stored in either Universal Transport Medium^TM^ (Copan) or RNeasy Lysis Buffer (Qiagen), see Appendix A Figure A2 for an overview. Nasal epithelial cells were cultured for immunological analyses, if the requested SARS-CoV-2 qPCR analysis was negative and the remaining brushes stored in RNeasy Lysis Buffer will be used for transcriptome, (epi)genome and microbiome analyses. Urine was collected in a container and distributed in 4 tubes with 2.8 mL of urine per tube for storage at −80 °Celsius (C) until metabolome analysis. Breath was stored in a Tenax^®^ sorbent tube and in an electronic nose (eNose) (Gerstel) tube that were stored in a fridge (2–8 °C) until further gas chromatography-mass spectrometry and eNose analysis respectively will be performed. ENose technology is a non-invasive approach that applies advanced pattern recognition algorithms for analysis of the mixture of volatile organic compounds (VOCs) in exhaled breath. For the eNose measurement patients were asked not to eat and drink, except for water, 2 h before the study visit and to not consume alcohol for 8 h prior to the study visit. Furthermore, exhaled breath was analyzed in real-time by the (eNose) using the SpiroNose^®^ (Breathomix). The bioelectric impedance analysis was performed after emptying of the bladder with a BODYSTAT 500 device.

Questionnaires

The following questionnaires were used to describe health characteristics of patients from a physical, fatigue, cognitive, psychological, selfcare and participation perspective. The Fatigue Severity Scale (FSS) is a validated questionnaire to assess fatigue on daily functioning in patients [50]. A score of ≥4 indicated that fatigue is influencing daily functioning. For patients that have a FSS score ≥4, the DePaul Symptom Questionnaire 2 (DSQ-2) was also completed. The DSQ-2 was developed to assess the symptomatology and case definition fulfillment of individuals with myalgic encephalomyelitis (ME) and chronic fatigue syndrome (CFS) [51]. The Hospital Anxiety and Depression Scale (HADS) is a validated questionnaire for screening anxiety and depression, which can be calculated separately [52,53]. The total score can range from 0 to 21 that can be interpreted as the following: scores up to seven means no anxiety or depression, scores from eight to ten are referred to as mild anxiety or depression and scores from eleven and up are considered as moderate to severe [53]. The Utrecht Scale for Evaluation of Rehabilitation Participations (USER-P) is a measurement tool to assess participation objectively as well as subjectively [54], covering experienced restrictions. A sum of scores on the three aspects was made and each was converted to a 0–100 scale, where higher scores indicate good levels of participation [54]. In this study a cut-off percentage, below which participation is not considered satisfactory, is 60%. The Checklist for cognitive consequences after an ICU admission (CLC-IC) was used to investigate the extent to which post-ICU COVID-19 patients experience cognitive impairment from their intensive care unit (ICU) admission. A score of ≥2 was taken as cut-off value for the experience of cognitive impairment from an ICU admission. In the EuroQol 5D-5L (EQ5D) questionnaire patients can score from having ‘no problems’ up to ‘extreme problems’ on mobility, selfcare, usual activities, pain/discomfort and anxiety/depression [55]. A cut-off score >1 per area was used to indicate problems. With the Patient-Reported outcome measurements information system (PROMIS) questionnaire, three global domains are measured, namely physical, mental and social aspects of health and well-being. PROMIS instruments are expressed using a T-score, where the value of 50 is assigned to the average score in a reference population [56]. The Primary Care PTSD Screen for DSM-5 (PC-PTSD-5) is designed to identify patients with PTSD symptoms [57]. Scores range from 0-5 with a cut-off score of 4 [57]. The Behavioral Regulation in Exercise Questionnaire-2 BREQ-2, is a questionnaire to measure self-determination with respect to motivation in physical exercise [58]. The Regulation of Eating Behaviors Scale (REBS) was used to assess the quality of motivation for healthy nutrition [59]. Finally, a short questionnaire on the presence of persistent symptoms is taken at baseline and is being sent to patients monthly throughout the follow-up of nine months.

Exposome

The Sniffer Bike is a real-time air quality monitor and was attached to an outside window of the participants’ home with three suction cups and measures the concentrations of outdoor particulate matter (PM1, M2.5, and PM10) over a six-month period [60]. Time-weighted personal PM2.5 exposure was measured using the UPAS, which the participant wore for 24 h at a time, with a keycord, for a total of 4 measurements to allow for seasonal variation. After these 4 monitoring periods, a questionnaire was taken to assess in what kind of environment the patient was located and if the UPAS was in close proximity to the patient during this period. In addition to these air quality measurements, silicone wristbands were deployed to assess exposure to organic particles, including volatile organic compounds [61]. Data on physical activity (e.g., steps, intensity), heart rate, and sleep quality was collected with GARMIN Vivosmart 4 activity trackers over a six-month period. Finally, questionnaires collected data on perceived environmental quality, and data from the Utrecht Exposome Hub will be used to assess long-term residential exposure to a variety of environmental factors, including ambient air pollution and green space. These extensive exposome data provide the unique opportunity to measure individual exposures in order to determine their effects on the development of long COVID.

Intervention
Personalized counselling intervention

The counselling approach was based on previous studies within the Maastricht University [62,63]. The intervention consisted of individual, group and educational sessions. The individual sessions (±30 min) took place once per month via telephone or digitally. The first individual sessions were an intake session (±45-60 min) during which subjects were asked to construct specific learning goals based on the physical activity level and dietary intake. Every month one of the researchers contacted the subject to discuss the progression of the learning goals using motivational interviewing. Additionally, two digital group sessions (±60 min) were offered for every subject during which subjects were stimulated to discuss their personal learning goals, share their experiences and share tips or strategies that they used. Furthermore, three 45-minute long digital educational sessions took place. During the educational sessions, professionals in the field of COVID-19 healthcare, rehabilitation and nutrition informed the patients with relevant insights and provided them with useful tips to improve their physical activity and dietary intake. Educational sessions were recorded and a record link was sent to subjects who were not able to attend the live sessions. The information gained during the educational sessions and the group sessions and the applicability of them were discussed for each subject during the individual sessions. Subjects that were not included in the intervention group (i.e. control subjects) were also invited to attend the educational sessions.

Nutritional support

The intervention group was voluntary provided with additional nutritional support (RemuneTM; Smartfish). The supplement (±230 kcal) is high in omega-3 fatty acids (2.0 g) including eicosapentaenoic acid (EPA; 1.2 g) and docosahexaenoic acid (DHA; 0.8 g) from fish oils, 25-hydroxy-vitamin D3 (10 µg) and whey protein concentrate (10 g). Previous studies have shown that this nutritional supplement has nutrition-related effects on inflammation, metabolic parameters and anorexia, which could also be affected in COVID-19 survivors [64,65,66]. The nutritional supplement was available in two flavours (peach and raspberry) and subjects could choose which flavours they wanted to receive. They were allowed to ingest both flavours to increase compliance. In general, subjects were advised to take one 200 mL supplement each day. Only in case of malnutrition, defined by the GLIM criteria, subjects were advised to take two supplements per day [67].

Qualitative analyses

The effectiveness of the personalized counselling intervention and the experiences of the subjects was evaluated using an evaluation questionnaire and a semi-structured in-depth interview via phone or digitally after completion of the full intervention. Subjects were asked to complete the evaluation questionnaire (±20 min) which consisted of statements regarding experiences of the individual, educational and group sessions. During the semi-structured in-depth interview (±1 h), additional open-ended questions were asked to gain more insight into the effectiveness of the intervention and the experiences of the subjects.

The end-questionnaire were analysed using descriptive statistics. Qualitative methods were used to analyse the semi-structured in-depth interview. In short, the interview will be transcribed verbatim and anonymized. A coding tree will subsequently be developed and the codes will be analysed using NVivo.

**Table A1:** Patient characteristics stratified by radiographic and pulmonary function test abnormalities without patient with pre-existing lung disease, including asthma, COPD and interstitial lung disease, N = 74. Patients were classified according to the number of persistent symptom categories (left) or the presence of radiological/radiological abnormalities. In case of missing data, it will be indicated of how many patients the data is available. Categorical variables are described as n (% of n), and continuous variables as median (interquartile range, (IQR)) or mean ± standard deviation depending on normality. BMI: Body Mass Index. COPD: Chronic obstructive pulmonary disease.

	No Pulmonary Function Test/Radiological Abnormalities (N = 16)	Pulmonary Function Test/Radiological Abnormalities (N = 57)
**General**		
Age (Years)	51.94 ± 5.80	54.14 ± 6.31
Female	9 (56.2%)	23 (39.7%)
**Smoking status**		
Current smoker	0 (0.0%)	3 (5.2%)
Ex-smoker	9 (56.2%)	29 (50.0%)
Never smoker	7 (43.8%)	26 (44.8%)
**BMI**		
Normal	2 (12.5%)	6/57 (10.5%)
Overweight	4 (25.0%)	25/57 (43.9%)
Obese	10 (62.5%)	26/57 (45.6%)
**Ethnicity**		
Caucasian	11/15 (73.3%)	41/52 (78.8%)
African	3/15 (20.0%)	4/52 (7.7%)
Asian	0/15 (0.0%)	2/52 (3.8%)
Latin-American	0/15 (0.0%)	2/52 (3.8%)
Other	1/15 (6.7%)	3/52 (5.8%)
**Level of education**		
Secondary education	0/13 (0.0%)	14/47 (29.8%)
Vocational education	8/13 (61.5%)	16/47 (34.0%)
Bachelor	4/13 (30.8%)	11/47 (23.4%)
Master	1/13 (7.7%)	6/47 (12.8%)
**Current comorbidities**		
Asthma	0/15 (0.0%)	0 (0.0%)
COPD	0/15 (0.0%)	0 (0.0%)
Cardiovascular disease	4/15 (26.7%)	20 (34.5%)
Heart failure	0/15 (0.0%)	3 (5.2%)
Deep vein thrombosis and pulmonary embolism	1/15 (6.7%)	5 (8.6%)
Diabetes	1/15 (6.7%)	11 (19.0%)
Hepatic disease	0/15 (0.0%)	5/57 (8.8%)
Renal Failure	1/15 (6.7%)	4/57 (7.0%)
Interstitial lung disease	0/15 (0.0%)	0 (0.0%)
Neurological disorders	0/15 (0.0%)	2 (3.4%)
Rheumatic diseases	2/15 (13.3%)	4 (6.9%)
**Acute COVID-19 characteristics**		
No hospitalization	4 (25.0%)	3 (5.2%)
Hospitalization	12 (75.0%)	55 (94.8%)
Intensive care admission	3 (18.8%)	17 (29.3%)
Duration of hospitalization	12; 6.50 (3.00, 8.25)	54; 10.50 (7.00, 16.00)
Thrombosis	2/15 (13.3%)	9/55 (16.4%)
Pulmonary embolism	1/15 (6.7%)	9/56 (16.1%)
WHO disease Severity (mild)	4 (25.0%)	3 (5.2%)
WHO disease Severity (moderate)	10 (62.5%)	41 (70.7%)
WHO disease Severity (severe)	2 (12.5%)	14 (24.1%)
**Oxygen supplementation and ventilation**		
Nasal cannula	6 (37.5%)	28 (48.3%)
Non-rebreating mask	3 (18.8%)	10 (17.2%)
Non-invasive ventilation	0 (0.0%)	2 (3.4%)
Invasive ventilation	2 (12.5%)	12 (20.7%)
Vaccination status		
No	5 (31.2%)	19 (32.8%)
Yes, 1 dose	6 (37.5%)	12 (20.7%)
Yes, 2 or more doses	5 (31.2%)	27 (46.6%)
**Pharmacological treatment during acute COVID-19**		
Antivirals	⅒ (10.0%)	0/50 (0.0%)
Convalescent plasma	0/7 (0.0%)	0/30 (0.0%)
Antibiotics	0/11 (0.0%)	0/51 (0.0%)
Immunosuppressives	11/12 (91.7%)	52/54 (96.3%)
Other	12/12 (100.0%)	48/53 (90.6%)
**Rest complaints**		
Fatigue	10 (62.5%)	33 (56.9%)
Respiratory	11 (68.8%)	43 (74.1%)
Neurological	11 (68.8%)	37 (63.8%)
Cardiovascular	4 (25.0%)	13 (22.4%)
Gastrointestinal	5 (31.2%)	12 (20.7%)
Other	5 (31.2%)	8 (13.8%)
**Dominant virus strains**		
Beta	4 (25.0%)	20 (34.5%)
Gamma	2 (12.5%)	8 (13.8%)
Delta	8 (50.0%)	24 (41.4%)
Omicron	2 (12.5%)	6 (10.3%)
**Blood tests**		
Hemoglobin (mmol/L)	11; 8.60 (8.45, 9.60)	39; 8.80 (8.30, 9.50)
Hematocrit (L/L)	9; 0.42 (0.42, 0.47)	33; 0.43 (0.40, 0.46)
Thrombocytes (10^9^/L)	9; 258.00 (243.00, 265.00)	36; 261.50 (206.00, 290.75)
Leukocytes (10^9^/L)	9; 6.00 (5.60, 7.70)	36; 6.45 (5.50, 7.98)
Eosinophiles (10^9^/L)	9; 0.10 (0.08, 0.18)	31; 0.12 (0.08, 0.17)
Basophiles (10^9^/L)	9; 0.03 (0.02, 0.05)	31; 0.04 (0.03, 0.06)
Neutrophiles (10^9^/L L)	9; 3.40 (2.98, 3.60)	28; 3.75 (2.65, 4.49)
Lymphocytes (10^9^/L)	9; 2.16 (1.63, 2.78)	31; 1.98 (1.73, 2.59)
Monocytes (10^9^/L)	9; 0.47 (0.40, 0.59)	31; 0.54 (0.43, 0.72)
Creatinine (μmol/L)	10; 72.50 (67.00, 80.75)	38; 81.50 (70.00, 89.00)
EGFR (mL/min/1.73 m^2^)	10; 88.50 (86.25, 90.00)	38; 86.50 (72.25, 90.00)
ALAT (U/L)	9; 23.00 (18.00, 29.00)	33; 27.00 (20.00, 37.00)
ASAT (U/L)	9; 22.00 (20.00, 29.00)	32; 23.00 (20.25, 27.00)
LDH (U/L)	9; 170.00 (159.00, 191.00)	33; 204.00 (179.00, 235.00)
CRP (mg/L)	9; 2.40 (1.00, 3.20)	34; 2.10 (1.33, 5.88)
Glucose (mmol/L)	6; 5.50 (4.75, 5.50)	27; 5.70 (5.35, 6.45)
Creatine Phosphokinase (U/L)	6; 91.00 (74.75, 104.25)	22; 96.50 (67.50, 199.50)
NTProBNP (ng/L)	10; 50.70 (50.00, 73.50)	29; 50.70 (50.00, 87.00)

At the Amsterdam UMC, Leiden UMC and Spaarne Gasthuis sample 1 is stored in Universal Transport Medium 1 mL and the other collected nasal samples are stored in 1 mL RNeasy Lysis Buffer. For VieCuri Medisch Centrum all collected nasal samples (1–4) are stored in cryotubes filled with 1 mL of RNeasy Lysis Buffer. Nasal samples that are collected in RNeasy Lysis Buffer are kept on ice until storage in a −80 °C freezer.
